# Primary abdominal hydatid cyst presenting in emergency as appendicular mass: a case report

**DOI:** 10.1186/1749-7922-4-13

**Published:** 2009-04-03

**Authors:** Utpal De

**Affiliations:** 1 Dept of Surgery, Medical College Hospital, 88, College Street, Kolkata 78, India

## Abstract

Hydatid disease, caused by Echinococcus granulosus, is a common parasitic infection of the liver. Disseminated intra-abdominal hydatid disease may occur following rupture of the hydatid cyst into the peritoneal cavity, producing secondary echinococcosis. Rarely cyst may develop de-novo in the peritoneal cavity without involvement of any other intra-abdominal organs. We present a unique case of 56-year-old woman with a primary intraabdominal hydatid cyst in the right iliac fossa masquerading as appendicular lump.

## Background

Hydatid disease (HD), caused by cestode Echinoccocus granulosus, is a significant health problem where animal husbandry is common. [1] Dogs or other carnivores are definitive hosts, whereas sheep or other ruminants are intermediate hosts. Man becomes an accidental intermediate host by ingestion of eggs which develop into cysts causing complication and even mortality (4%). [1,2] Common sites include liver (75%) and lungs (15%). [1] Peritoneal echinococcosis (13%) is usually secondary. [2] Primary peritoneal echinococcosis is rare. [2] Primary peritoneal hydatid cyst presenting as an appendicular lump is unique.

## Case presentation

A 56 year old lady presented with acute right lower abdominal pain, nausea, vomiting and fever since five days. Abdominal examination revealed a tender firm mass in the right iliac fossa, measuring 5 cm × 3 cm, with restricted mobility. Muscle guarding was present over the lump. Straight leg rising, cough sign and rebound tenderness were positive. Further investigations were conducted to address clinical suspicion of appendicular mass.

**Figure 1:**
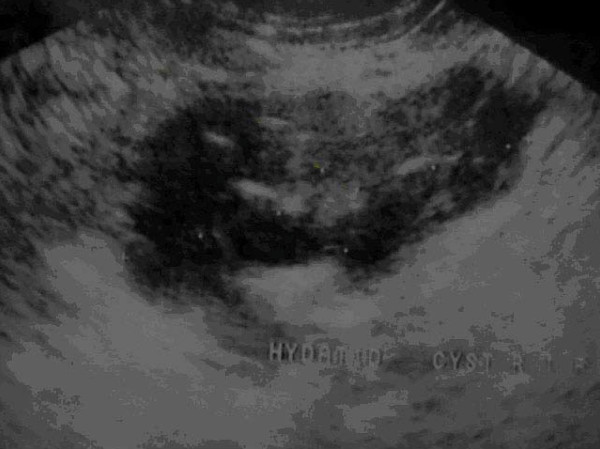
**USG showing Hydatid cyst in the right iliac fossa**.

Laboratory investigations revealed a haemoglobin level of 9.2 g/dL, neutrophilic leucocytosis (16,000/mm3) and marked eosinophilia (19%). Ultrasonography (USG) abdomen revealed a multiseptated cyst (5.2 cm × 2.5 cm) with honeycomb appearance in the right iliac fossa, suggestive of HD (Figure 1). Rest of the abdomen did not reveal any other hydatid cyst. ELISA (enzyme-linked immunosorbent assay using purified *Echinococcus *antigen, positive with a titre of more than 1:128.) for hydatid was positive.

At laparotomy the cyst was found to be located in the appendicular mesentry. Excision and appendectomy was performed. Other areas of the abdomen did not reveal any cysts. Recovery was uneventful and patient was discharged with Albendazole (800 mg/day) for one month. The patient is doing well after one year follow-up. Repeat abdominal USG after one year follow-up was within normal limits.

## Discussion

Intraperitoneal hydatid cysts usually develop secondary to spontaneous or iatrogenic rupture of hepatic, splenic, or mesenteric cysts. Rarely isolated primary cyst may develop in the peritoneum without evidence of cysts in other intra abdominal organs.

Primary peritoneal echinococcosis accounts for 2% of all abdominal hydatidosis. [2] Dissemination occurs either by lymphatic [3] or systemic [4] circulation. Clinical manifestations are due to mass effect of enlarging abdominal cyst.

Diagnosis is confirmed by radio-imaging studies (abdominal sonography and computerized tomography) complimented with serological tests (Complement fixation test, Indirect hemagglutination test and ELISA). [5,6]

Primary peritoneal hydatid cyst masquerading as ovarian, mesenteric, duplication and other intra-abdominal cysts have been reported. All these patients had evidence of hydatosis in other peritoneal organs. [1-8] A single primary peritoneal hydatid cyst without any hepatic or extrahepatic organ involvement mimicking appendicular lump has been unheard of as yet.

Surgery is the treatment of choice for primary abdominal HD. [7,8]

Pre operative courses of Albendazole should be considered in order to sterilize the cyst, decrease the chance of anaphylaxis, decrease the tension in the cyst wall (thus reducing the risk of spillage during surgery) and to reduce the recurrence rate post-operatively. [7,8] Intra-operatively, the use of hypertonic saline or 0.5% silver nitrate solutions before opening the cavities tends to kill the daughter cysts and therefore prevent further spread or anaphylactic reaction. [7,8]

A recurrence rate of 2% [8] and survival rate of 95% has been reported in patient undergoing operative intervention. [8] The efficacy of Albendazole, as sole medical therapy results in successful treatment in up to 40% of cases. [7,8]

## Conclusion

Suspicion of the disease in endemic areas aids prompt diagnosis and treatment. Hydatid cyst masquerading as appendicular lump has not been reported so far.

## Consent

Written informed consent was obtained from the patient for publication of this case report and any accompanying images. A copy of the written consent is available for review by the Editor-in-Chief of this journal.

## Competing interests

The author declares that they have no competing interests.
